# *Streptomyces bathyalis* sp. nov., an actinobacterium isolated from the sponge in a deep sea

**DOI:** 10.1007/s10482-021-01528-4

**Published:** 2021-02-17

**Authors:** Chandra Risdian, Wiebke Landwehr, Manfred Rohde, Peter Schumann, Richard L. Hahnke, Cathrin Spröer, Boyke Bunk, Peter Kämpfer, Peter J. Schupp, Joachim Wink

**Affiliations:** 1 Microbial Strain Collection (MISG), Helmholtz Centre for Infection Research (HZI), 38124 Braunschweig, Germany; 2 Research Unit for Clean Technology, Indonesian Institute of Sciences (LIPI), 40135 Bandung, Indonesia; 3 Partner Site Hannover-Braunschweig, German Centre for Infection Research (DZIF) , Braunschweig, Germany; 4 Central Facility for Microscopy, Helmholtz Centre for Infection Research (HZI), 38124 Braunschweig, Germany; 5 Leibniz Institut DSMZ-German Collection of Microorganisms and Cell Cultures, 38124 Braunschweig, Germany; 6 Institut für Angewandte Mikrobiologie, Justus-Liebig-Universität Giessen, 35392 Giessen, Germany; 7 Institute for Chemistry and Biology of the Marine Environment, Carl von Ossietzky University of Oldenburg, 26129 Oldenburg, Germany

**Keywords:** Deep‐sea sponge, The North Atlantic Ocean, Polyphasic taxonomy, *Streptomyces*

## Abstract

A novel actinobacterium, designated ASO4wet^T^, was isolated from the unidentified sponge (SO4) in the deep sea collected of the North Atlantic Ocean. Study of 16S rRNA gene sequences indicated that strain ASO4wet^T^ is a member of the genus *Streptomyces* and showed the closest similarities to *Streptomyces karpasiensis* K413^T^ (98.87 %), *Streptomyces glycovorans* YIM M 10366^T^ (98.38 %), and *Streptomyces abyssalis* YIM M 10400^T^ (97.53 %). Strain ASO4wet^T^ contained MK-9(H8) as the predominant menaquinone and the major fatty acids are iso-C_16:0_, anteiso-C_15:0_, and iso-C_15:0_. Polyphasic taxonomy was carried out between strain ASO4wet^T^ and its phylogenetically closely related *Streptomyces* strains, which further elucidated their relatedness and revealed that strain ASO4wet^T^ could be distinguished from currently known *Streptomyces* species. Strain ASO4wet^T^ clearly represents a novel species in genus *Streptomyces*. We propose the name *Streptomyces bathyalis* sp. nov., with the type strain ASO4wet^T^ (= DSM 106605^T^ = NCCB 100657^T^). Analysis of the whole-genome sequence of *S. bathyalis* revealed that genome size is 7,377,472 bp with 6332 coding sequences.

## Introduction

In the effort of finding new bioactive compounds from novel *Streptomyces*, some studies recently are focused on the neglected and unexplored regions in order to enlarge the successful isolation of new species (Goodfellow et al. 2017). The deep sea is one of the underexplored areas on Earth. One of the reasons is probably because of its extreme environments such as high pressure, low temperature, less oxygen concentration, and lack of light intensity. More than 30,000 marine natural products have been isolated and about 2 % of those are from deep-sea organisms, including Actinobacteria from the genus *Streptomyces, Marinactinospora*, and *Verrucosispora* (Tortorella et al. 2018).

Previously, some strains and species such as *Streptomyces* sp. NTK 937 (Hohmann et al. 2009), *Streptomyces olivaceus* FXJ8.012 (Liu et al. 2013), *Streptomyces* sp. SCSIO 04496 (Luo et al. 2015), *Streptomyces indicus* (Luo et al. 2011), and *Streptomyces nanhaiensis* (Tian et al. 2012) were reported to be isolated from the deep-sea sources. *Streptomyces nanhaiensis* was found in the northern South China Sea at 1632 m below sea level (Tian et al. 2012), while *Streptomyces indicus* was isolated from the Indian Ocean depth of 2434 m (Luo et al. 2011).

*Streptomyces* is a genus of aerobic Gram-positive bacteria and one of its characters is the morphology that contains substrate and aerial mycelia (Williams et al. 1983). A minor amount of species of this genus was reported having no aerial mycelia such as *Streptomyces somaliensis* (Brumpt 1906) Waksman and Henrici 1948 (Approved Lists 1980) (Skerman et al. 1980) and *Streptomyces sudanensis* (Quintana et al. 2008). *Streptomyces* is one of the genera from Actinobacteria and many of them are isolated from soil (Ritacco et al. 2003; Risdian et al. 2018); however, in some previous studies, they are also reported to be found in the rhizosphere of the plant (Xiao et al. 2009), mangrove sediment (Handayani et al. 2018), and marine sediment (Xu et al. 2012). *Streptomyces* is one of the important producers of antibiotics, considering that more than half of the antibiotics used nowadays are produced by this group of bacteria (Lucas et al. 2013). However, they are mainly terrestrial strains (Kemung et al. 2018).

In the course of our investigation of Actinobacteria from the deep sea in the extended Continental shelf of Portugal, near Madeira Islands, strain ASO4wet^T^ was isolated from an unidentified sponge (SO4) collected by ROV (remotely operated vehicle) from the North Atlantic Ocean (36°15.19038 N, 14°32.99767 W) at 1092 m water depth.

## Materials and methods

### Actinobacteria isolation and morphological study

The isolation of actinobacteria was performed using 5336-ASW medium (soluble starch 10.0 g, casein 1.0 g, K_2_HPO_4_ 0.5 g, MgSO_4_.7H_2_O 5.0 g, artificial seawater (ASW) 1000 ml, agar 20.0 g, pH 7.3) and incubated at 30 °C. Artificial seawater (ASW) contained 3.9 % (w/v) of sea salt from ATI Coral Ocean. Morphological observations of spores and mycelia on International *Streptomyces* Project 2 or ISP2 agar (yeast extract-malt extract), ISP3 agar (oatmeal), ISP4 agar (inorganic salt- starch) agar, ISP5 agar (glycerol-asparagine), ISP6 agar (peptone-yeast extract-iron), and ISP7 agar (tyrosine) (Shirling and Gottlieb 1966) at 30 °C for 14 days. The colours of mycelium (aerial and substrate) and diffusible pigments were evaluated by comparison with the RAL-code (https://www.ral-farben.de) (Charousová et al. 2015). Spore chain morphology and spore-surface ornamentation of strain ASO4wet^T^ were observed after growing on ISP 3 agar medium (Shirling and Gottlieb 1966) for 4 weeks at 30 °C by Zeiss Merlin field emission scanning electron microscope (SEM) (Landwehr et al. 2018).

## Physiological and biochemical studies

Growth of strain ASO4wet^T^ at different temperatures (15, 20, 25, 30, 37 and 44 °C) on GYM medium (glucose-yeast extract-malt extract) and pH range (pH 2, 3, 4, 5, 6, 7, 8, 9 and 10) on ISP2 medium were evaluated after incubation for 14 days. Utilisation of carbohydrate was examined on ISP9 medium supplemented with 1 % carbon sources (Shirling and Gottlieb 1966), the sodium chloride tolerance was investigated as described by Kutzner (1981), and the enzymatic activity profile analysis was conducted by using API ZYM strips (Humble et al. 1977). Antibiotic susceptibility was investigated by the disc-diffusion plate method (Bauer et al. 1966) using antibiotic discs on ISP2 agar medium incubated for 7 days at 30 °C. Eight antibiotic discs were used: ampicillin (10 µg/disc), erythromycin (15 µg/disc), gentamycin 30 (µg/disc), tetracycline (30 µg/disc), vancomycin (30 µg/disc), cefotaxime (30 µg/disc), rifampicin (5 µg/disc), and penicillin G (6 µg/disc).

## Phylogenetic analysis

Extraction of DNA, PCR amplification, and purification of the 16S rRNA gene sequence was performed as described by Landwehr et al. (2016). PCR amplified templates were sequenced using a 96-capillary-system from Applied Biosystems (ABI), a 3730xl DNA Analyzer. Sequence data were compiled with the BioEdit program (Hall 1999) (http://www.mbio.ncsu.edu/BioEdit/bioedit.html). The almost-complete 16S rRNA gene sequence (1,417 nucleotides) of strain ASO4wet^T^ was obtained and submitted for BLAST analysis (Altschul et al. 1990) (https://blast.ncbi.nlm.nih.gov/Blast.cgi). The 16S rRNA gene sequence was deposited in GenBank as MT036271. The similarity and homology of the 16S rRNA gene sequence were examined for sequence homology with the database of 16S rRNA gene sequences from the National Center for Biotechnology Information (NCBI) (https://www.ncbi.nlm.nih.gov/). The 16S rRNA gene sequences of strain ASO4wet^T^ and some related type strains were aligned using the CLUSTAL W algorithm (Thompson et al. 1994) from the MEGA X software package (Kumar et al. 2018). Phylogenetic analyses were performed using the maximum-likelihood (Felsenstein 1981) algorithm from MEGA X (Kumar et al. 2018). The topologies of the inferred trees were examined by bootstrap analyses (Felsenstein 1985) based on 1000 replicates. The resulting phylogenetic trees were rooted using the 16S rRNA gene sequence of *Actinospica robiniae* GE134769^T^ (AJ865863).

## Chemotaxonomy

Biomass for the chemical analyses was collected by cultivation in glucose-yeast-malt extract (GYM) medium in flasks on a rotary shaker (160 revolutions per minute) at 30 °C for 3–7 days. The freeze-dried cells from biomass were used for chemical analysis. The whole-cell diaminopimelic acid isomers and sugars were evaluated based on the method of Staneck and Roberts (1974). Menaquinones were extracted as described by Minnikin et al. (1984) and were analysed by high-performance liquid chromatography (Wink et al. 2017) equipped with diode-array detection and mass spectrometry (HPLC-DAD-MS). High-resolution electron spray ionisation mass spectrometry (HR-ESI-MS) data were recorded on a MaXis ESI-TOF-MS spectrometer (Bruker) equipped with an Agilent 1260 series RP-HPLC system using XBridge C18 column 2.1 × 100 mm, 1.7µm. Solvent A was isopropanol and solvent B was acetonitrile. The gradient system was 100 % B for 5 min, 35 % B in 5 to 15 min, and 50 % B in 16–20 min with the flow rate was 0.6 mL/min. The temperature of the column was 40 °C and the UV-detection was at 270 nm. The molecular formula of menaquinones was calculated using the Smart Formula algorithm, including the isotopic pattern (Bruker). The polar lipids were extracted according to Minnikin et al. (1977) and identified by two-dimensional thin-layer chromatography as described previously by Collins and Shah (1984).

Fatty acids were extracted, methylated and analysed using the Sherlock Microbial Identification (MIDI) system and the ACTIN version 6 database (Sasser 1990). For matrix-assisted linear desorption/ionisation-time-of-flight mass spectrometry (MALDI-TOF MS) analysis, the isolate ASO4wet^T^ was incubated at 30 °C for 6–8 days. The samples were prepared using ethanol/formic acid extraction, as described by Schumann and Maier (2014).

## DNA-DNA hybridisation and ribotyping analysis

DNA–DNA hybridisation was performed based on the method of Ziemke et al. (1998), except that for nick translation, 2 µg DNA was labelled during 3 h of incubation at 15 °C. This method was carried out for the DNA of strain ASO4wet^T^ and the strain *Streptomyces karpasiensis* DSM 42068^T^, *Streptomyces glycovorans* DSM 42021^T^, and *Streptomyces abyssalis* DSM 42024^T^. Standardised and automated ribotyping analysis was conducted using the RiboPrinter system (Hygiena) involving *Pvu*II as a restriction enzyme (Bruce 1996; Schumann and Pukall 2013).

## DNA extraction and complete genome sequencing

The complete genome sequence of strain ASO4wet^T^ was obtained via a combination of long-read PacBio and short-read Illumina-Sequencing. Therefore, DNA was isolated using Qiagen Genomic-tip 100/G (Qiagen, Hilden Germany) according to the instructions of the manufacturer. SMRTbell™ template library was prepared according to the instructions from PacificBiosciences, Menlo Park, CA, USA, following the Procedure & Checklist – Greater Than 10 kb Template Preparation. Briefly, for preparation of 15 kb libraries 8µg genomic DNA was sheared using g-tubes™ from Covaris, Woburn, MA, USA according to the manufacturer´s instructions. DNA was end-repaired and ligated overnight to hairpin adapters applying components from the DNA/Polymerase Binding Kit P6 from Pacific Biosciences, Menlo Park, CA, USA. Reactions were carried out according to the manufacturer´s instructions. For the bacterial DNAs, BluePippin™ Size-Selection to greater than 4 kb was performed according to the manufacturer´s instructions (Sage Science, Beverly, MA, USA). Conditions for annealing of sequencing primers and binding of polymerase to purified SMRTbell™ template were assessed with the Calculator in RS Remote, PacificBiosciences, Menlo Park, CA, USA. 1 SMRT cell was sequenced on the PacBio *RSII* (PacificBiosciences, Menlo Park, CA, USA) taking one 240-minutes movie.

Bacterial DNAs libraries for sequencing on Illumina platform were prepared to apply Nextera XT DNA Library Preparation Kit (Illumina, San Diego, USA) with modifications according to Baym et al. (2015). Samples were sequenced on NextSeq™ 500.

## Genome assembly and annotation

Genome assembly performed applying the RS_HGAP_Assembly.3 protocol included in SMRT Portal version 2.3.0 using default parameters. The assembly revealed a single linear chromosome with a coverage value of 117x. Error-correction was performed by a mapping of Illumina short reads onto finished genome using Burrows-Wheeler Alignment bwa 0.6.2 (Li and Durbin 2009) in paired-end (sample) mode using default settings with subsequent variant and consensus calling using VarScan 2.3.6 (Koboldt et al. 2012) Automated genome annotation was carried out using the NCBI Prokaryotic Genome Annotation Pipeline PGAP (Tatusova et al. 2016). The assembly was also uploaded to RAST (Rapid Annotation using Subsystem Technology) server (https://rast.nmpdr.org/) (Aziz et al. 2008) and antiSMASH server (https://antismash.secondarymetabolites.org/) (Medema et al. 2011; Blin et al. 2019) for metabolic reconstruction analysis and prediction of secondary metabolite gene clusters, respectively. The complete genome sequence of strain ASO4wet^T^ was deposited at NCBI GenBank under accession number CP048882 in the NCBI Genome database (https://www.ncbi.nlm.nih.gov/genome).

## Results and discussion

**Fig. 1:**
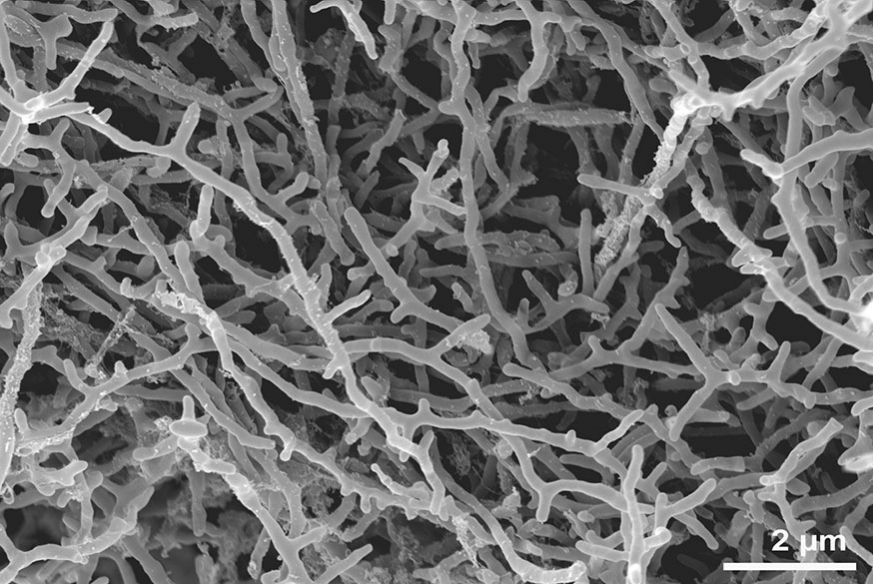
Scanning electron micrographs of aerial mycelium with no spore detected of strain ASO4wet^T^ after incubation on ISP 3 agar for 4 weeks at 30 °C

Strain ASO4wet^T^ was found to grow well on ISP2, ISP3, ISP4, and ISP5, while sparse in ISP6 and ISP7 (Table 1). The aerial mycelium can be seen in ISP3 and ISP7. The diffusible pigment was not detected on all tested medium. The strain formed aerial mycelium, albeit no spore was detected on ISP3 agar (Fig. 1).

**Table 1:** Characteristics of strain ASO4wet^T^ on various ISP agar media after incubation for 14 days at 30 °C

Agar medium	Growth	Substrate mycelium colour	Aerial mycelium colour	Soluble pigment
Yeast extract-malt extract (ISP2)	Good	Light ivory	None	None
Oatmeal (ISP3)	Good	Light ivory	Grey white	None
Inorganic salt- starch (ISP4)	Good	Ivory	None	None
Glycerol-asparagine (ISP5)	Good	Light ivory	None	None
Peptone-yeast extract-iron (ISP6)	Sparse	Sandy yellow	None	None
Tyrosine (ISP7)	Sparse	Nutbrown	Light grey	None

Strain ASO4wet^T^ grew on medium ISP2 at 15-37^o^C (optimum at 25-30^o^C) and at pH 6–9 (optimum at pH 7). The strain grew on CYE medium (10 g casein peptone l^− 1^, 5 g yeast extract l^− 1^, 20 g agar l^− 1^, pH 7) supplemented with up to 10 % NaCl. Antibiotic susceptibility test indicated that the strain was sensitive to ampicillin, erythromycin, gentamycin, penicillin G, tetracycline, vancomycin, and rifampicin. However, it was resistant to cefotaxime (30 µg/disc).

**Fig. 2:**
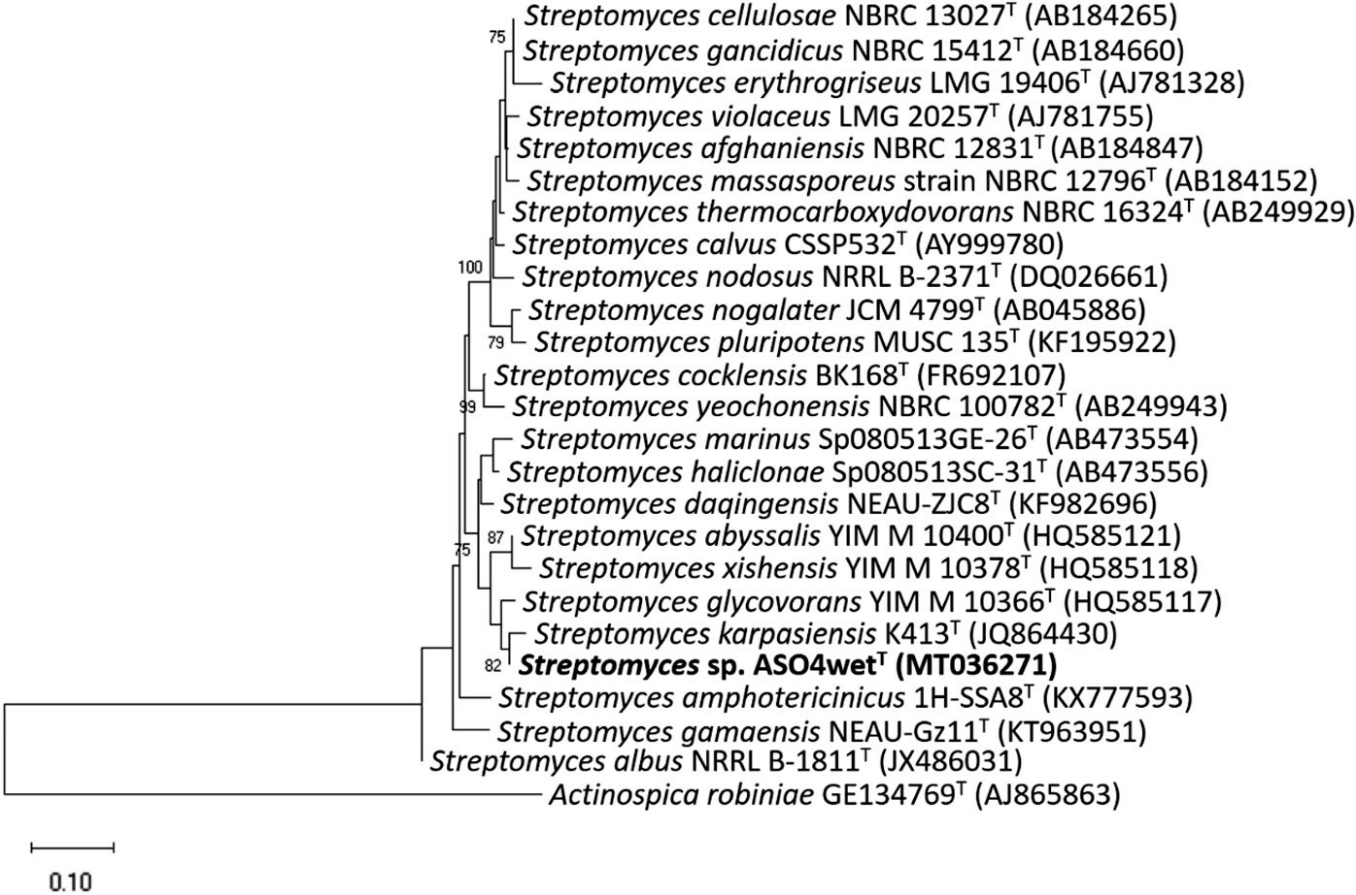
Maximum-likelihood tree based on 16S rRNA gene sequences (1408 positions in the final dataset) showing relationships between strain ASO4wet^T^ and the related type strains of *Streptomyces* species. The phylogenetic trees were rooted using the 16S rRNA gene sequence of *Actinospica robiniae* GE134769^T^ (AJ865863). The evolutionary distances were computed using the Tamura-Nei method (Tamura and Nei 1993). Numbers at the nodes are percentage bootstrap values with 1,000 replicates, only values above 70 % are shown. Bar 0.10 substitutions per nucleotide position

According to the result from the NCBI server, isolate ASO4wet^T^ related to the genus *Streptomyces*. The strain closely related to *Streptomyces karpasiensis* K413^T^ (98.87 %), *Streptomyces glycovorans* YIM M 10366^T^ (98.38 %), and *Streptomyces abyssalis* YIM M 10400^T^ (97.53 %). Strain ASO4wet^T^ formed a stable clade with *Streptomyces karpasiensis* K413^T^ that was supported by 82 % bootstrap value in the maximum-likelihood tree based on the 16S rRNA gene sequence (Fig. 2).

**Fig. 3:**
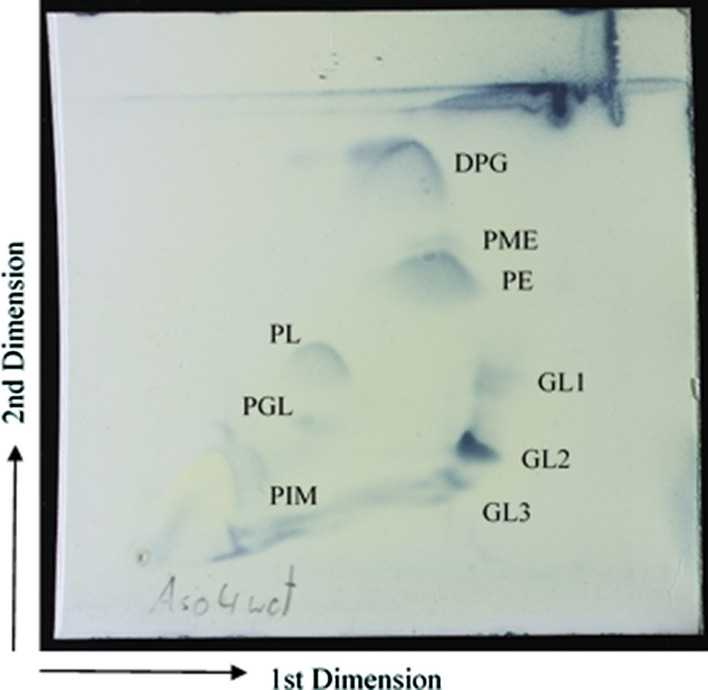
Polar lipid observed in strain ASO4wet^T^. DPG: diphosphatidylglycerol; PME: phosphatidyl-N-methyl-ethanolamine; PE: phosphatidylethanolamine; PL: unknown phospholipid; GL1-3: unknown glycolipid; PGL: unknown phosphoglycolipid; PIM: phosphatidylinositol mannoside

Cell-wall hydrolysates of strains ASO4wet^T^ contained LL-diaminopimelic acid, which is suggested that it belongs to cell-wall type I (Lechevalier and Lechevalier 1970). Whole-cell hydrolysates of strains ASO4wet^T^ contained glucose and xylose. The major fatty acids of strain ASO4wet^T^ were iso-C_16:0_ (35. 1 %), anteiso-C_15:0_ (22 %), iso-C_15:0_ (13.8 %), anteiso-C_17:0_ (8.8 %), and iso-C_14:0_ (6.3 %). The menaquinone composition was identified as MK-9(H8) and MK-9(H6) in a ratio of 12:1. The polar lipids were identified as diphosphatidylglycerol, phosphatidylglycerol, phosphatidylethanolamine, phosphatidyl-N-methyl-ethanolamine, phosphatidylinositol mannoside, and four unidentified polar lipids (Fig. 3). These chemotaxonomic properties of strain ASO4wet^T^ had similar profiles to some species from the genus *Streptomyces* that have been reported previously (Kämpfer et al. 2008; Busarakam et al. 2014; Ayed et al. 2018).

**Fig. 4:**
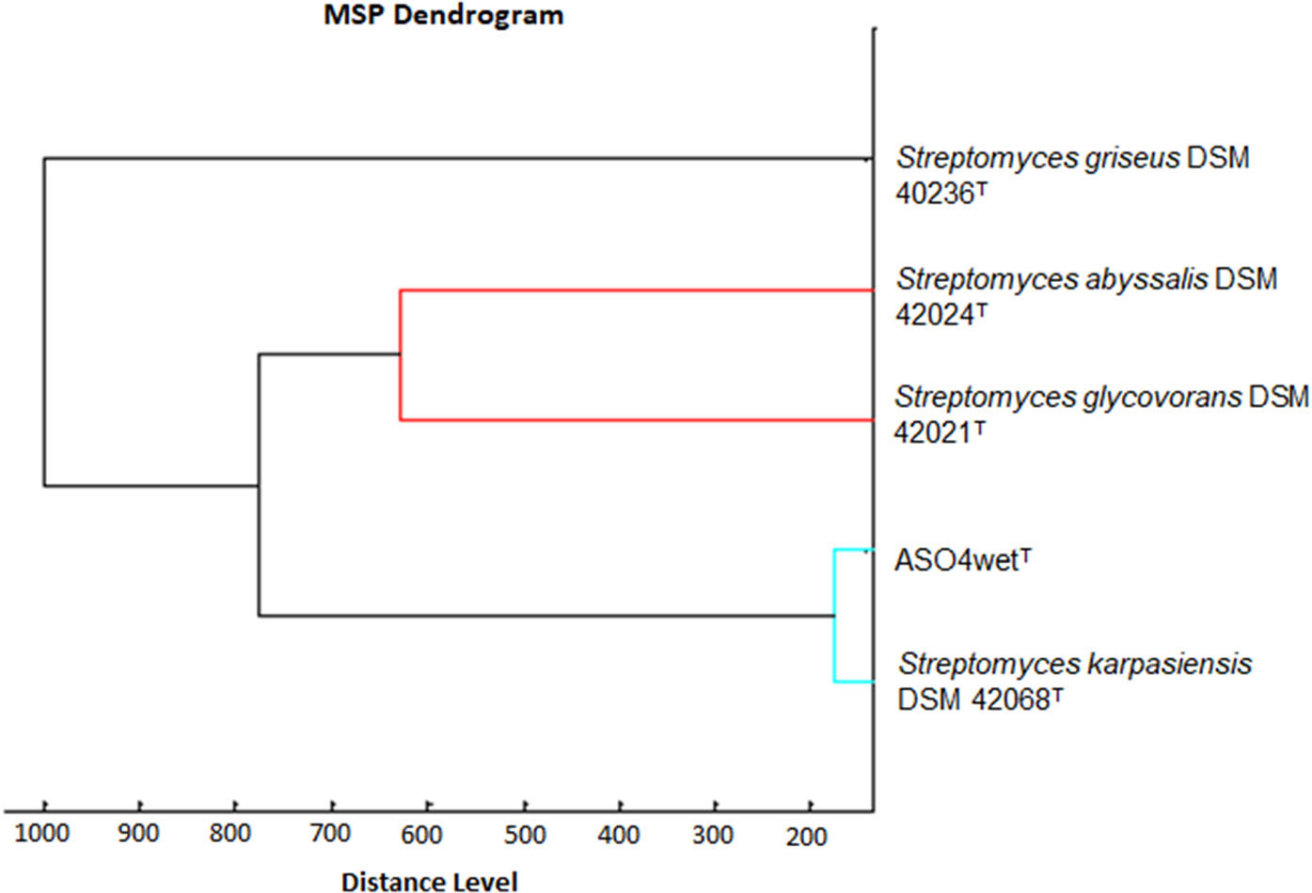
MALDI-TOF dendrogram of the strain ASO4wet^T^ and its most closely related strains. *Streptomyces* *griseus* DSM 40236^T^ was used as an outgroup

**Fig. 5:**
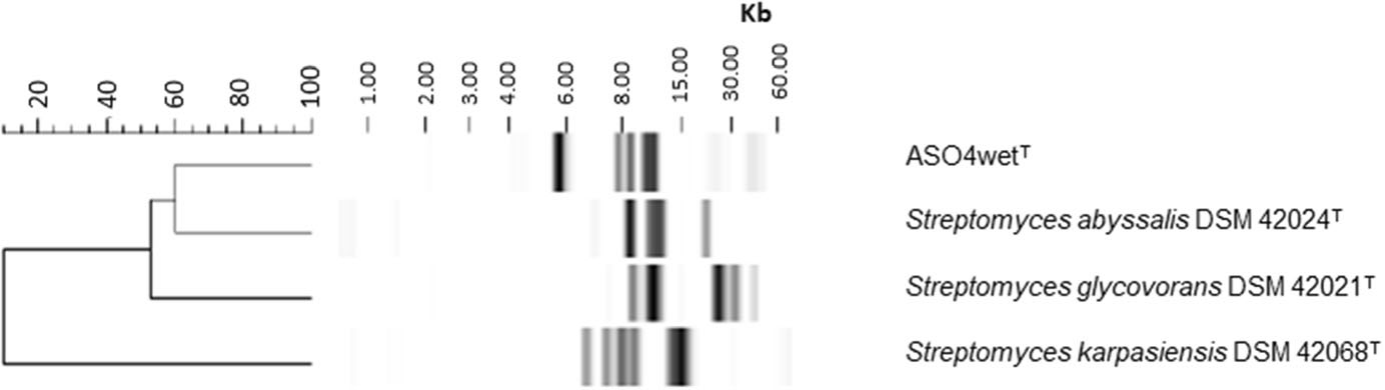
Dendrogram of RiboPrinter® patterns (restriction enzyme *Pvu*II, BioNumerics Software) of the strain ASO4wet^T^ and its closely related *Streptomyces* strains

The MALDI-TOF analysis result also suggested that strain ASO4wet^T^ formed a clade with *Streptomyces karpasiensis* DSM 42068^T^ (Fig. 4). The comparison of the fingerprints using the BioNumerics software (version 7.6.1; Applied Maths, Belgium) exhibited the differences between strain ASO4wet^T^, *Streptomyces karpasiensis* DSM 42068^T^, *Streptomyces glycovorans* DSM 42021^T^, and *Streptomyces abyssalis* DSM 42024^T^ (Fig. 5). All strains displayed different band patterns.

To determine whether strain ASO4wet^T^ represent a novel species, DNA-DNA hybridisation (DDH) was conducted to further delineate the relatedness between strain ASO4wet^T^ and its closely related type strains, i.e., *Streptomyces karpasiensis* DSM 42068^T^, *Streptomyces glycovorans* DSM 42021^T^, and *Streptomyces abyssalis* DSM 42024^T^. The levels of DNA-DNA relatedness between strain ASO4wet^T^ and *Streptomyces karpasiensis* DSM 42068^T^, *Streptomyces glycovorans* DSM 42021^T^, and *Streptomyces abyssalis* DSM 42024^T^ were 40.4/54.7 %, 40.5/44.4 %, and 35.8/28.2 % respectively. These values are below the threshold value of 70 %, as suggested by Wayne et al. (1987) for determining novel species for bacterial strains.

**Fig. 6:**
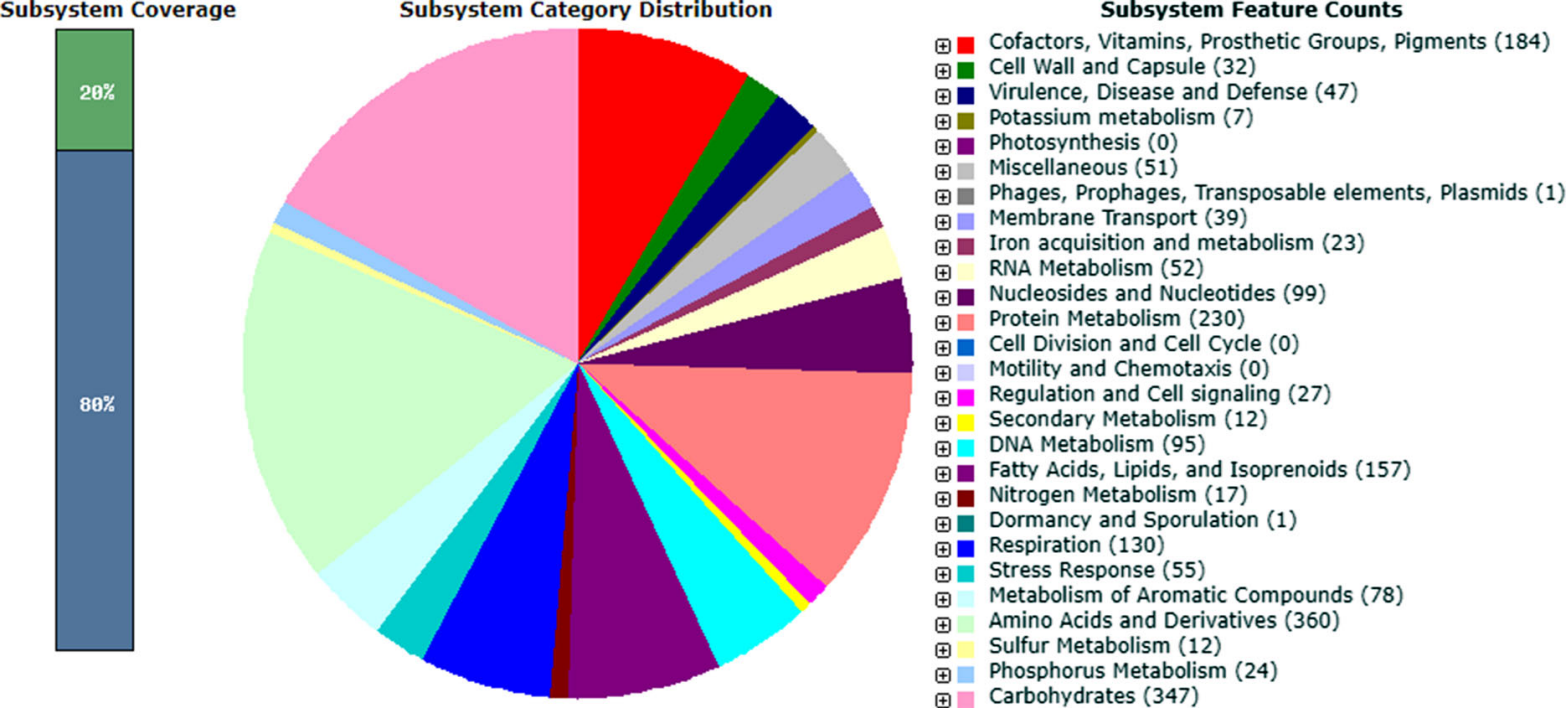
Subsystem category distribution of strain ASO4wet^T^ based on RAST annotation server (https://rast.nmpdr.org/)

Genome sequencing of strain ASO4wet^T^ resulted in a single linear chromosome typical for members of the genus *Streptomyces* consisting of 7,377,472 bp. The G + C content was 70.24 mol%. 6,332 coding sequences, 59 tRNA genes, and six rRNA operons were found after NCBI PGAP annotation. Analysis by using RAST server revealed that only 20 % of the annotated genes were assigned to subsystems (Fig. 6). Among the subsystem categories present in the genome, amino acids and derivatives metabolism had the highest feature counts (360), followed by carbohydrates metabolism which had 347 feature counts. However, only one feature count detected for dormancy and sporulation, which is different from the some other *Streptomyces* strains that have at least 10 feature counts (Busarakam et al. 2014; Ser et al. 2018; Quinn et al. 2020). The antiSMASH server predicted 23 secondary metabolite biosynthesis gene clusters, with six clusters showed more than 60 % similarities to known biosynthetic gene clusters: hopene biosynthetic gene cluster (61 %), planosporicin biosynthetic gene cluster (100 %), geosmin biosynthetic gene cluster (100 %), isorenieratene biosynthetic gene cluster (62 %), ectoine biosynthetic gene cluster (100 %), and desferrioxamine E biosynthetic gene cluster (100 %).

Besides the result of genotypic studies such as 16S rRNA gene analysis and DNA-DNA hybridisation, strain ASO4wet^T^ can also be discriminated from its closely related type strains by some phenotypic properties (Table 2). Lipase (C14) activity could not be observed for strain ASO4wet^T^, whereas *Streptomyces glycovorans* DSM 42021^T^ was positive. There was the β-galactosidase activity for strain ASO4wet^T^, while in the all compared type strains, it was not detected. Strain ASO4wet^T^ had no β-glucosidase activity, while all of the tested type strains possessed it. Strain ASO4wet^T^ exhibited good growth on the ISP 9 medium supplemented with arabinose, while *Streptomyces karpasiensis* DSM 42068^T^ showed no growth. Phosphatidylinositol mannoside was detected in strain ASO4wet^T^ but not in *Streptomyces karpasiensis* DSM 42068^T^ based on data previously reported by Veyisoglu et al. (2014).

**Table 2:** Phenotypic properties that distinguish strain ASO4wet^T^ from the most closely related *Streptomyces* species

Characteristics	1	2	3	4
Esterase (C4)	+	(+)	(+)	+
Lipase (C14)	−	−	+	−
Trypsin	+	+	+	−
Chymotripsin	+	+	+	−
α-galactosidase	−	−	−	−
β-galactosidase	+	−	−	−
β-glucoronidase	-	−	(+)	(+)
α-glucosidase	+	+	+	+
β-glucosidase	−	(+)	+	+
Glucose	+	+	+	+
Arabinose	+	−	++	+
Sucrose	+	+	++	++
Xylose	++	++	++	++
Inositol	++	+	++	++
Mannose	(+)	+	−	+
Fructose	(+)	+	−	+
Rhamnose	+	+	−	+
Raffinose	++	+	++	++
Cellulose	−	+	−	−
Polar lipids	DPG, PG, PE, PME, PIM, PGL, 3 GLs	DPG, PG, PE, PME, PI, 3 PLs, 2 PGLs, 2 GLs*	DPG, PG, PME, PE, PIM, PI, 6PLs**	DPG, PG, PME, PIM, PI, 5PLs**
Predominant Menaquinone	MK-9(H8)	MK-9(H8)*	MK-9(H8)**	MK-9(H4), MK-9(H6), MK-9(H8)**

Strains: 1, ASO4wet^T^; 2, *Streptomyces karpasiensis* DSM 42068^T^ (= K413^T^); 3, *Streptomyces glycovorans* DSM 42021^T^(= YIM M 10366^T^); 4, *Streptomyces abyssalis* DSM 42024^T^ (= YIM M 10400^T^)

++ better growth; + positive result or good growth; - negative result or no growth; (+) weakly positive result or weak growth. DPG, diphosphatidylglycerol; PG, phosphatidylglycerol; PE, phosphatidylethanolamine; PME, phosphatidyl-N-methyl-ethanolamine; PI, phosphatidylinositol; PIM, phosphatidylinositol mannoside; PL, unknown phospholipid; PGL, unknown phosphoglycolipid; GL, unknown glycolipid. *Data from Veyisoglu et al. (2014); ** data from Xu et al. (2012)

In conclusion, strain ASO4wet^T^ represents a novel species in the genus *Streptomyces*, for which the name *Streptomyces bathyalis* sp. nov. is proposed.

### Description of *Streptomyces bathyalis* sp.nov.

*Streptomyces bathyalis* (ba.thy.al’is. L. neutrum substantive from the Greek bathys (deep) the part of the pelagic zone between 1,000 and 4,000 m).

Aerobic, Gram-positive actinomycete that forms branched substrate mycelium. Aerial hyphae can be seen only in ISP3 and ISP7. Spores are not detected in any medium tested even after 4 weeks of incubation at 30 °C. It grows well on ISP2, ISP3, ISP4, and ISP5 after 2 weeks incubation at 30 °C. Optimum growth occurs at 25–30 °C and at pH 7. The NaCl tolerance is 0–10 % (w/v) NaCl. Positive for esterase (C4), trypsin, chymotrypsin, β-galactosidase, and α-glucosidase. Negative for lipase (C14), α-galactosidase, β-glucuronidase, and β-glucosidase. Glucose, arabinose, sucrose, xylose, inositol, mannose, fructose, rhamnose, raffinose are used as sole carbon sources, but not cellulose. Major fatty acids are iso-C16:0 (35.0 %), anteiso-C15:0 (22.0 %), and iso-C15:0 (13.8 %). The major menaquinone is MK-9(H8). The diagnostic amino acid in the peptidoglycan is LL-diaminopimelic acid. Glucose and xylose are present in whole-cell hydrolysates. The type strain is ASO4wet^T^ (= DSM 106605^T^ = NCCB 100657 ^T^), isolated from a sponge collected from the North Atlantic Ocean at 1092 m depth. The genomic DNA G + C content of the type strain is 70.24 mol%. The genome size is 7,377,472 bp with 6,332 coding sequences, 59 tRNA genes, and six rRNA operons. The complete genome and the 16S rRNA sequence of strain ASO4wet^T^ were deposited at NCBI GenBank with accession number CP048882 and MT036271, respectively.

## Data Availability

The GenBank accession number for the 16S rRNA gene sequence of strain ASO4wet^T^ is MT036271. The GenBank accession number for complete genome of strain ASO4wet^T^ is CP048882.
